# Genome-wide identification and expression profile of *HIR* gene family members in *Oryza sativa* L

**DOI:** 10.3389/fpls.2024.1492026

**Published:** 2024-11-19

**Authors:** Jiahao Li, Lingyu Shao, Qian Wang, Liyu Shi, Wei Wu, Wei Chen, Zhenfeng Yang, Saisai Li

**Affiliations:** Zhejiang Key Laboratory of Intelligent Food Logistic and Processing, College of Biological and Environmental Sciences, Zhejiang Wanli University, Ningbo, China

**Keywords:** rice, HIR, gene family, expression profiles, abiotic stress

## Abstract

The *hypersensitive-induced reaction* (*HIR*) gene family is associated with the hypersensitive response (HR) in plant defense against pathogens. Although rice (*Oryza sativa* L.) is a crucial food crop, studies on its *HIR* genes are limited. This study identified six *HIR* members, categorized into three phylogenetic clades. The analyses of phylogenetics, gene structures, and collinear relationships revealed a high conservation of these genes, featuring the stomatin/prohibitin/flotillin/HflK/C domain. *OsHIR* genes were regulated by *cis*-acting elements, including ARE, SARE, DRE, LTR, and GARE. *OsHIRs* were widely expressed in multiple plant organs, including roots, stems, and leaves. These genes respond to various abiotic stresses (like drought and low temperature) and hormone treatments (including ABA, SA, GA, and MeJA) with overlapping yet distinct expression patterns. Our results indicate that *OsHIRs* are involved in abiotic stresses and hormone responses, which provides a basis for further functional analysis of OsHIRs in rice crop plants.

## 1 Introduction

Plants suffer various kinds of biotic and abiotic stresses during growth. They developed effector-triggered immunity (ETI) and PAMP-triggered immunity (PTI) as defense system against pathogens (Jones and Dangl, 2006). ETI can trigger a hypersensitive response (HR), which restricts pathogen spread via programmed cell death (Block and Alfano, 2011; Greenberg and Yao, 2004; Jones and Dangl, 2006). Hypersensitive-induced reaction (HIR) proteins, homologs of *Nicotiana tabacum*’s NG1, cause HR-like lesions upon overexpressed (Karrer et al., 1998). Three proteins from maize (*Zea mays*), namely ZmHIR1, ZmHIR2, and ZmHIR3, were discovered as the first NG1 homologs (Nadimpalli et al., 2000). Many additional *HIR* genes have since been discovered according to DNA and amino acid similarities (Chen et al., 2007; Hakozaki et al., 2004; Rostoks et al., 2003; Yu et al., 2008, 2013; Chen et al., 2017; Zhao et al., 2019; Li et al., 2022).

The HIR protein belongs to the proliferation, ionization, and death (PID) superfamily and features the stomatin/prohibitin/flotillin/HIK (SPFH) structural domain, essential for ion channels regulation, cell proliferation, and apoptosis. Numerous PID family members are known to be associated with lipid rafts, forming membrane microdomains (Nadimpalli et al., 2000). Predominantly, HIR proteins localize to the plasma membrane (Choi et al., 2011; Duan et al., 2013; Qi et al., 2011; Wang et al., 2024; Li et al., 2019; Zhao et al., 2019; Zhou et al., 2010), with a minor portion found in the nucleus, extracellular matrix, tonoplast, nuclear membrane, and mitochondria, as reported by various studies (Li et al., 2022, 2019; Zhao et al., 2019; Choi et al., 2011; Zhou et al., 2010).

HIR proteins are vital for plant stress responses. In wheat (*Triticum aestivum* L.), the expression levels of *TaHIR1* and *TaHIR3* were reduced when exposed to temperature, drought, and high salt stresses, as well as with exogenous abscisic acid (ABA) and ethrel treatments (Duan et al., 2013). Studies have demonstrated that methyl jasmonate (MeJA) treatment elevated *MdHIR4* transcript level in apple (Zhao et al., 2019). The identification of phytohormone response elements, specifically the abscisic acid response element (ABRE), within the promoter region of the *BnHIR* gene indicates its involvement in ABA response (Li et al., 2022). Additionally, *HIR* genes have been implicated in the plant’s defense mechanisms against a range of pathogens, including fungi (Yu et al., 2008; Wang et al., 2024; Sun et al., 2021), bacteria (Zhou et al., 2010; Choi et al., 2011; Zhao et al., 2019), and viruses (Mei et al., 2020; Li et al., 2019).

Rice is a crucial economic crop in the world with a rich history of farming and consumption. However, our knowledge of the OsHIR family remains limited. In this research, we identified six OsHIR-encoding genes within the *Oryza sativa* genome and conducted bioinformatics analyses, expression profiling, and assessments of their responses to abiotic stresses and hormone treatments. Additionally, we performed subcellular localization analysis to confirm OsHIR protein functions. Our findings contribute crucial insights into the functions of OsHIR protein in rice.

## 2 Materials and methods

### 2.1 Sequence retrieval and date sources

The HIR amino acid sequences of *Aabidopsis thaliana*, *Triticum aestivum*, *Hordeum vulgare*, and *Zea mays* were obtained from the NCBI website. These sequences were then utilized as queries in a protein BLAST search against rice (*Oryza sativa* spp. *japonica*) RefSeq Protein (FASTA). The *OsHIR* genes were identified based on their chromosomal location in rice and by a BLAST comparison using the *Arabdopsis* Information Resource (https://www.arabidopsis.org/). The amino acid numbers, isoelectric points (theoretical pI), molecular weights, and subcellular localizations of the identified OsHIR protein candidates were predicted by ProtParam (https://www.expasy.org/resources/protparam) and GenScript WoLF PSORT tools (https://www.genscript.com/wolf-psort.html/).

### 2.2 Phylogenetic and synteny analyses

The phylogenetic analysis of HIR proteins were conducted in software MEGA 11 using the Maximum Likelihood method, following the approach described by Yoshida and Nei (2016). Synteny relationship analysis was performed using Advanced Circos integrated with TBtools for handling and visualizing large-scale datasets across the whole genome (Chen et al., 2023).

### 2.3 Structural characterization analysis

The *OsHIR* gene structures were assessed through CDD-search, and the conserved motifs of OsHIR proteins were found via the MEME tool (https://meme-suite.org/meme/). Results from these analyses were shown by TBtools.

### 2.4 Prediction of *cis*-acting elements in the promoters

The promoter sequences (2.0 kb) of *OsHIR* genes were obtained from the rice gene annotation file. Regulatory elements within the promoter regions were examined via PlantCARE (Rombauts et al., 1999; Lescot et al., 2002), and the results were displayed using the Simple BioSequence Viewer in TBtools.

### 2.5 Subcellular localization analysis

*OsHIR* cDNA was amplified through RT-PCR and inserted into pCAMBIA1301-GFP plasmid using specific primers listed in **Supplementary Table S1**. *Agrobacterium* cells containing 35S::OsHIRs-GFP or 35S::GUS-GFP (control) were injected into tobacco leaves. GFP fluorescence was captured in the FITC (EGFP) channel via a confocal laser microscope system (Nikon A1+, Tokyo, Japan) 60 hours post-infiltration.

### 2.6 Plant material, growth conditions and treatments

‘N1P’ rice (*Oryza sativa* spp. *japonica*) seedlings were cultivated in a glasshouse with a 16-h light/8-h dark photoperiod at 28-30°C and 60% humidity. Roots, stems, and leaves from four-week-old plants were rapidly frozen in liquid nitrogen and stored at -80°C for RT-qPCR analysis.

The rice seedlings experienced low temperature stress at 4°C and drought stress with 10% PEG6000. For phytohormone treatments, seedlings were sprayed with 100 μM abscisic acid (ABA), 2 mM salicylic acid (SA), 100 μM gibberellin (GA), or 100 μM methyl jasmonate (MeJA) with 0.02% (v/v) Tween 20, while control seedlings were treated with Tween 20 only. Leaf samples were collected at designated time points and stored at -80°C.

### 2.7 RT-qPCR analysis

RNA extraction, reverse transcription, and quantitative PCR (qPCR) were conducted as previously detailed (Li et al., 2019). The gene-specific primers used in this study was listed in **Supplementary Table S1**.

### 2.8 Statistical analysis

Statistical analyses were performed using SPSS 22.0 software (SPSS Inc., Chicago, IL, USA). Results were presented as mean ± standard error and evaluated using Student’s t-test (* *p*≤ 0.05, ** *p*≤ 0.01, *** *p*≤ 0.001).

## 3 Results

### 3.1 Genome-wide identification and naming of *OsHIR* members

**Figure 1:**
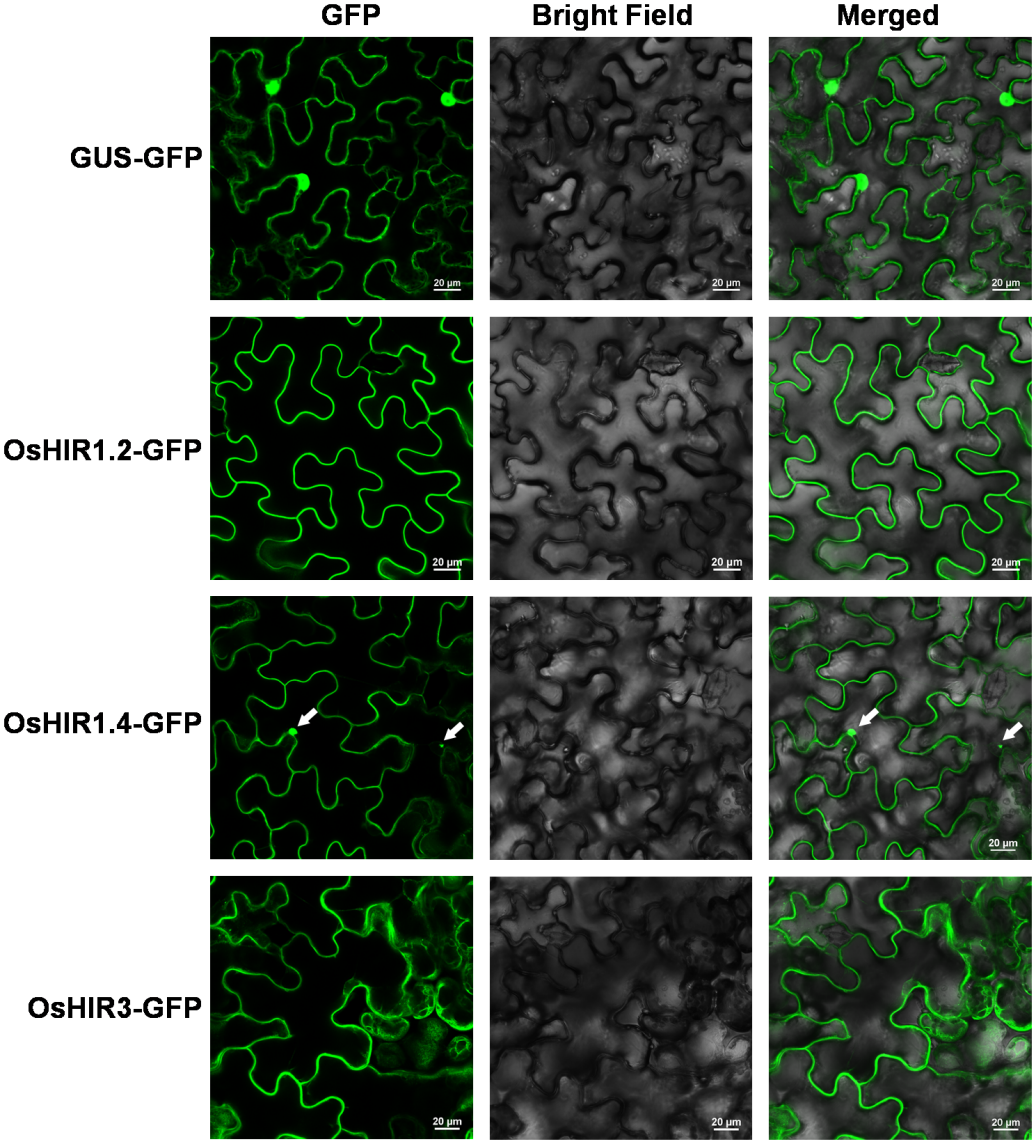
Subcellular localization of OsHIR proteins in *Nicotiana benthamiana* leaf epidermal cells. Arrow indicates the cytoplasmic aggregates. Scale bar: 20 μm.

To identify the *HIR* genes in rice (*Oryza sativa* L.), the amino acid sequences of three *Arabidopsis* HIR proteins (AtHIR1.1, AtHIR1.2, and AtHIR3) were served as queries for Protein BLAST against the *Oryza sativa* L. Genome Database. Six HIR-encoding genes, namely *OsHIR1.1*, *OsHIR1.2*, *OsHIR1.3*, *OsHIR1.4*, *OsHIR1.5*, and *OsHIR3*, were identified. These *OsHIR* genes were located on distinct chromosomes, as shown in **Table 1**; **Supplementary Figure S1**. The OsHIR proteins ranged from 284 (OsHIR1.3) to 314 (OsHIR1.1) amino acids, with molecular weights (MW) spanning 31379.85 (OsHIR1.3) to 33406.48 (OsHIR1.1) Da and isoelectric points (pI) from 5.21 (OsHIR1.4) to 6.54 (OsHIR1.2) (**Table 1**). The subcellular localization prediction analysis indicated that most OsHIR proteins were localized to the plasma membrane and cytoplasm, except OsHIR3, which localized to the plasma membrane, and OsHIR1.2 localized to both the nucleus and cytoskeleton (**Table 1**). To validate these predictions, four OsHIR proteins were transiently expressed in tobacco (*N. benthamiana*) leaf cells. The GFP signals of OsHIR1.2 and OsHIR3 proteins were distinctly visible in the plasma membrane, whereas the OsHIR1.4-GFP fusion protein signal was observed in both the plasma membrane and cytoplasmic aggregates (**Figure 1**).

**Table 1:** Physicochemical property and subcellular localization of OsHIRs in *Oryza sativa*.

Gene Name	Gene ID	Chromosome Localization	Amino Acid number	Molecular Weight (Da)	Theoretical pI	Subcellular localizaton
*OsHIR1.1*	Os01g0588100	Chr01: 22905349-22908006	314	33406.48	5.43	Plasma membrane/ Cytosol
*OsHIR1.2*	Os05g0591900	Chr05: 29486797-29492171	302	33043.74	6.54	Cytoskeleton/ Nucleus
*OsHIR1.3*	Os08g0398400	Chr08: 19011814-19015995	284	31379.85	5.22	Cytosol/ Cytoskeleton
*OsHIR1.4*	Os09g0361200	Chr09: 11796015-11799012	287	31606.07	5.21	Cytosol/ Cytoskeleton
*OsHIR1.5*	Os10g0464000	Chr10: 17117279..17120140	292	32125.96	5.81	Cytosol/ Nucleus
*OsHIR3*	Os06g0136000	Chr06: 1909658..1913036	288	32140.83	5.44	Plasma membrane/ Endoplasmic reticulum

### 3.2 Phylogenetic analysis

**Figure 2:**
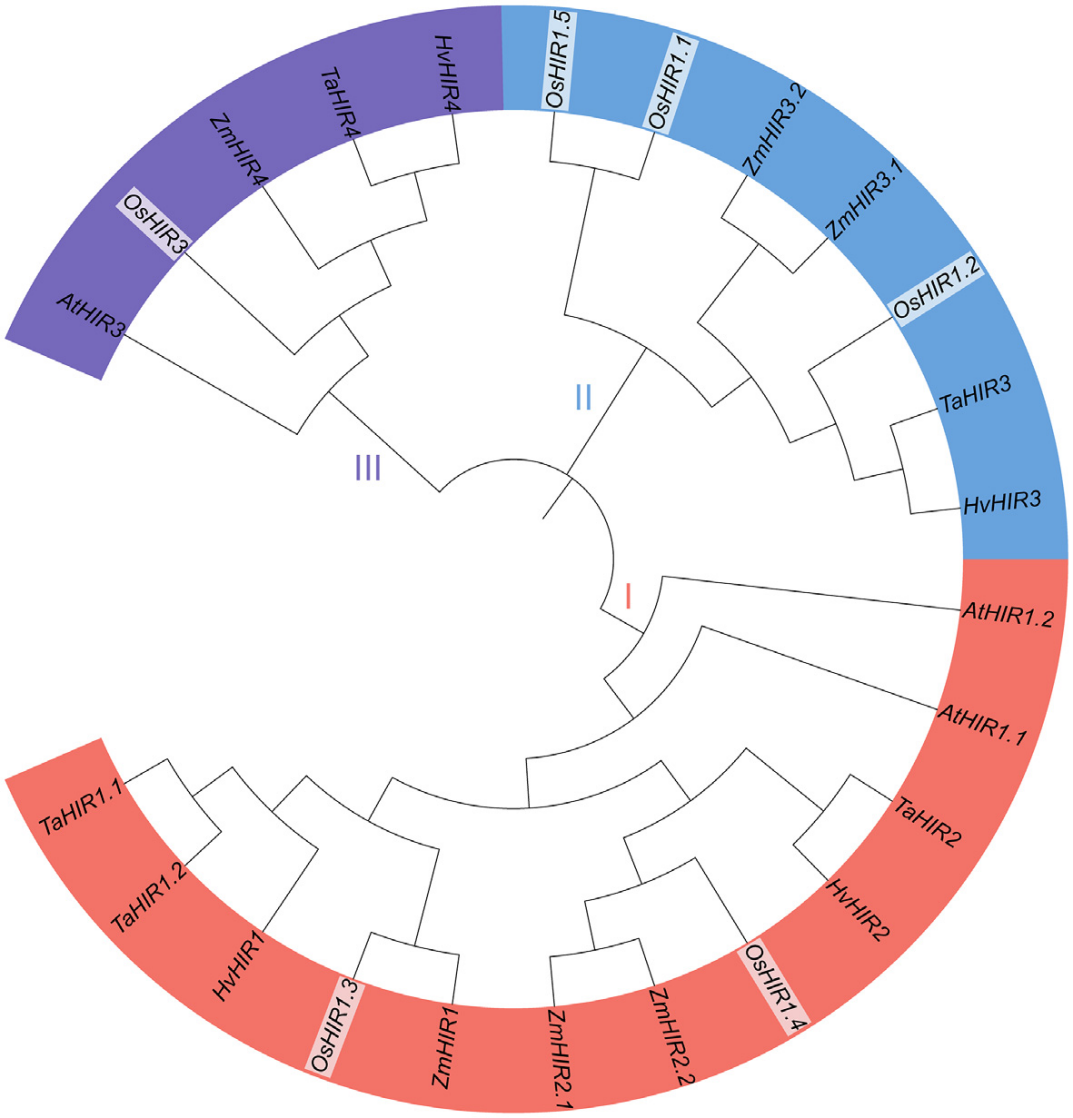
Phylogenetic analysis of HIR proteins among five plant species. The numbers on branches are bootstrap values calculated from 1000 replicates. AtHIRs, HIRs of *Arabidopsis*; OsHIRs, HIRs of *Oryza sativa* (rice); ZmHIRs, HIRs of *Zea mays* (maize); TaHIRs, HIRs of *Triticum aestivum* (wheat); HvHIRs, HIRs of *Hordeum vulgare* (barley). Clade I (red), II (blue), and III (purple) present the three phylogenetic groups.

To explore the evolutionary relationship of OsHIR proteins, the amino acid sequences of 24 HIR proteins from *Arabidopsis*, *Triticum aestivum* (wheat), *Zea mays* (maize), *Hordeum vulgare* (barley), and *Oryza sativa* (rice) were analyzed to build a phylogenetic tree. These proteins were grouped into three clades: clade I, which included two OsHIR proteins (OsHIR1.3 and OsHIR1.4); clade II, which consisted of three OsHIR proteins (OsHIR1.1, OsHIR1.2, and OsHIR1.5); and clade III, which had a single OsHIR3 protein (**Figure 2**). Furthermore, synteny relationship analysis of *OsHIR* genes revealed collinearity among four out of the six *OsHIRs* (**Supplementary Figure S1**).

**Figure 3:**
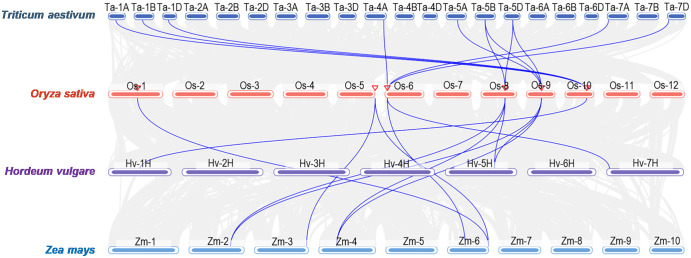
Syntenic analysis of *HIR* genes between *Oryza sativa* (rice) and other plant species including *Zea mays* (maize), *Triticum aestivum* (wheat), and *Hordeum vulgare* (barley).

To further clarify their evolutionary relationships, syntenic maps between *Oryza sativa* and *Zea mays*, *Triticum aestivum*, and *Hordeum vulgare* were constructed. 11, 8, and 4 homologous *HIR* gene pairs were shown between *O. sativa* and *T. aestivum*, *Zea mays*, and *H. vulgare*, respectively (**Figure 3**), whereas no pairs were found between *A. thaliana* and *O. sativa* (**Supplementary Figure S2**). These results indicated a closer evolutionary link between *O. sativa* and *T. aestivum* and *Zea mays*.

### 3.3 Conserved motifs, gene structure, and domains analyses

**Figure 4:**
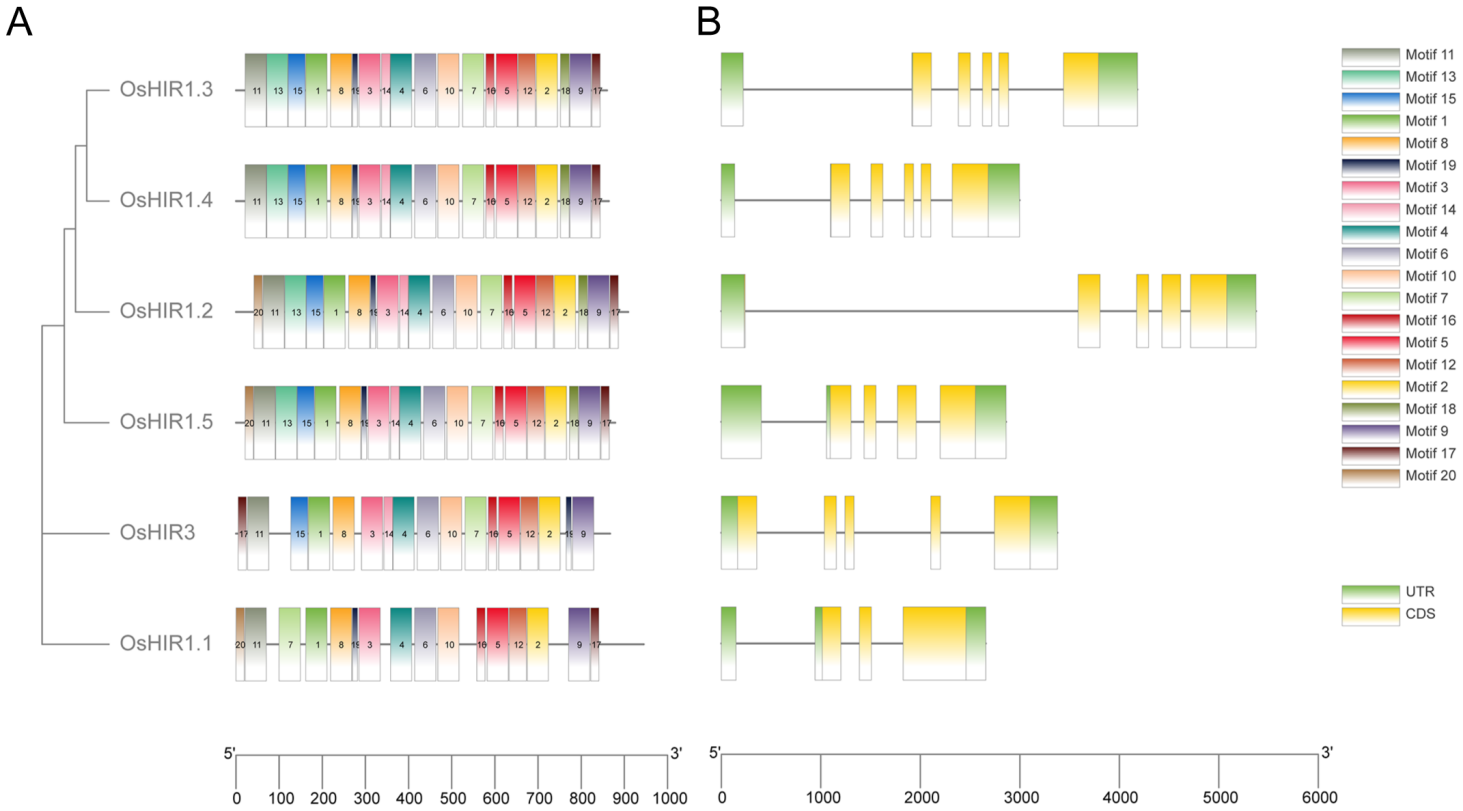
Analysis of conserved motifs and gene structure of *Oryza sativa HIR* genes. **(A)** Motif distribution of *OsHIRs*. Motifs from 1 to 20 are marked by different colors. **(B)** Gene structure analysis of *OsHIRs*. Untranslated regions, exons, and introns are shown as light green boxes, yellow boxes and horizontal lines, respectively.

To elucidate the evolution of OsHIR members, MEME analysis identified 20 conserved motifs. OsHIR1.2 and OsHIR1.5 contain all 20 predicted motifs, while OsHIR1.3 and OsHIR1.4 possess 19 motifs except motif 20. OsHIR1.1 contains 16 motifs, and OsHIR3 contains 17 motifs, excluding motif 13, 18, and 20 (**Figure 4A**). The gene structures of *OsHIR* are largely conserved, typically featuring 3-5 introns and 2-3 exons (**Figure 4B**). Notably, all OsHIR proteins contained the typical conserved SPFH domain, categorized under the PID superfamily (**Supplementary Figure S3**), indicating evolutionary conservation of functional domains among OsHIR proteins.

### 3.4 Prediction of *cis*-acting elements in promoters

**Figure 5:**
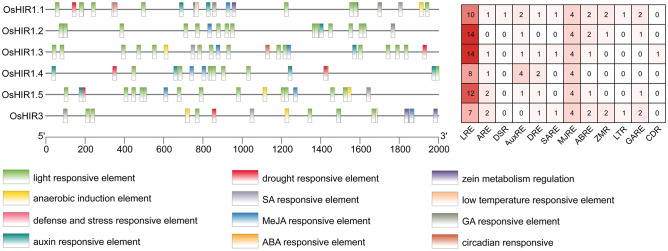
*Cis*-acting elements in the promoter regions of *OsHIR* genes. Different *cis*-acting elements in the promoters of *OsHIRs* were presented with differently colored boxes. The numbers of *cis*-acting elements was shown in the right. LRE, light responsive element; ARE, anaerobic induction element; DSR, defense and stress responsive element; AuxRE, auxin responsive element; DRE, drought responsive element; SARE, SA responsive element; MJRE, MeJA responsive element; ABRE, ABA responsive element; ZMR, zein metabolism regulation; LTR, low temperature responsive element; GARE, GA responsive element, CDR, circadian responsive.

To investigate the roles of *OsHIR* genes, the promoter analysis was conducted. A total of 244 *cis*-acting elements, including 12 significant ones, with 48% being light responsive, were identified (**Figure 5**). Furthermore, 5 of these elements were linked to plant hormones, while 3 were related to abiotic stress responses, highlighting the importance of *OsHIR* genes in hormone and stress responses in plants (**Figure 5**).

### 3.5 Expression patterns of *OsHIR* genes in various tissues

**Figure 6:**
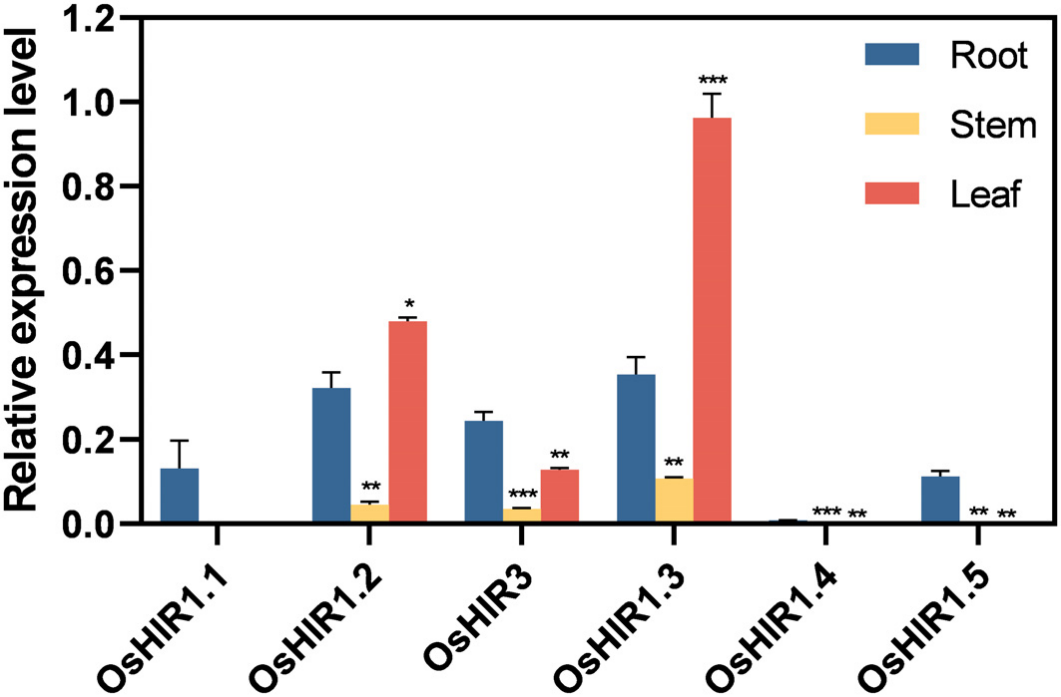
Expression profiles of *OsHIR* genes in different tissues. Data are mean ± SE (n = 3). The housekeeping gene *OsActin* was used as the reference transcript, which was set up as ‘1’. Asterisk represents a significant difference between the root and other tissues (*p< 0.05, **p< 0.01, and ***p< 0.001). Three independent experiments were conducted for experimental reproducibility.

The expression pattern of six *OsHIR* members across various tissues (root, stem, leaf) was analyzed using RT-qPCR. *OsHIR3*, *OsHIR1.2*, and *OsHIR1.3* were expressed highly in the leaf, while the expression of *OsHIR1.1*, *OsHIR1.4*, and *OsHIR1.5* was very low or undetectable in the stem and leaf (**Figure 6**).

### 3.6 Expression profiles under abiotic stress and hormonal treatment

**Figure 7:**
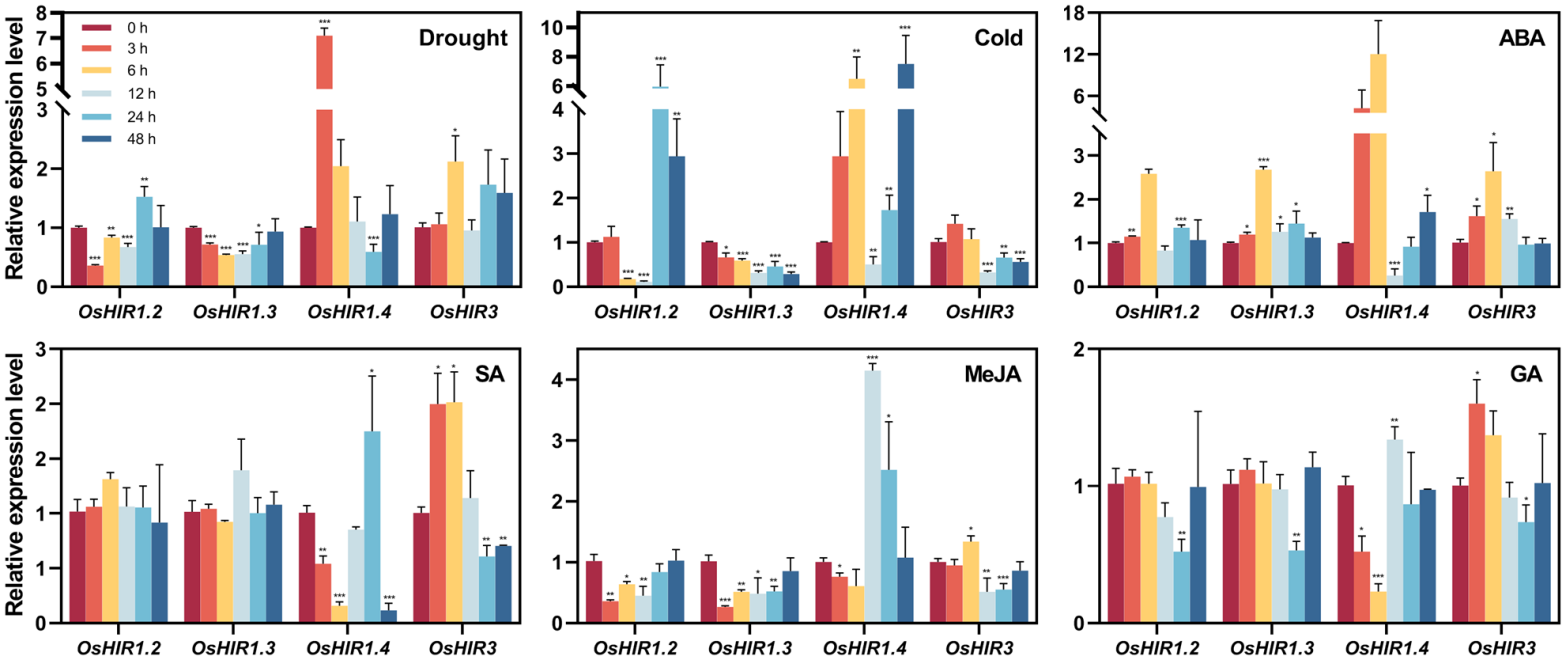
Expression profiles of *OsHIR* genes in response to different abiotic stress and plant hormone treatments. Four-week-old rice seedling were sprayed with 10% PEG6000, low temperature, 100 μM ABA, 100 μM GA, 100 μM MeJA or 2 mM SA with 0.02%(v/v) Tween 20. Plants sprayed with 0.02%(v/v) Tween 20 were used as mock control. After treatments, leaves were collected at 0, 3, 6, 12, 24, and 48h. Gene expression was normalized to control unstressed expression level, which was set up as ‘1’. The housekeeping gene *OsActin* was used as the reference transcript. Data are mean ± SE (n = 3). Asterisk represents a significant difference between the control and different treatment (*p< 0.05, **p< 0.01, and ***p< 0.001). Three independent experiments were conducted for experimental reproducibility.

Plants activate stress response genes when exposed to abiotic stress. Here, the transcription levels of four representative *OsHIR* members in rice treated with PEG6000, low temperature, ABA, GA, SA, or MeJA over varying durations were analyzed. Following PEG treatment, *OsHIR3* expression level increased twofold at 6 h, while *OsHIR1.2* and *OsHIR1.3* were down-regulated at 3, 6, and 12 h. *OsHIR1.2* was first down-regulated then up-regulated under cold stress, whereas *OsHIR3* and *OsHIR1.3* remained down-regulated. In contrast, *OsHIR1.4* exhibited initial up-regulation followed by down-regulation under drought and cold stress (**Figure 7**).

Most *OsHIR* genes were up-regulated by ABA but down-regulated by GA and MeJA. *OsHIR1.4* expression was inhibited at 12 h post-ABA treatment, showing an initial down-regulation followed by up-regulation under GA and MeJA. Notably, *OsHIR3* expression doubled at 3 and 6 h following SA treatment, remained stable at 12 h, and fell at 24 and 48 h, while *OsHIR1.2* and *OsHIR1.3* remained unchanged. *OsHIR1.4* showed significant inhibition at 3, 6, and 48 h, while it was up-regulated at 24 h (**Figure 7**). These findings reveal that *OsHIR* gene expression varies in response to abiotic stress and hormonal treatments.

## 4 Discussion

Hypersensitive-induced reaction (HIR) proteins, part of the PID superfamily, have been shown to be involved in plant defense responses (Nadimpalli et al., 2000). The number of *HIR* genes varies among species, reflecting the evolution of this gene family. Currently, researchers have identified four *HIR* genes in *Arabidopsis* (Qi et al., 2011), three in maize (*Zea mays*) (Nadimpalli et al., 2000), four in barley (*Hordeum vulgare*) (Rostoks et al., 2003), five in wheat (*Triticum aestivum*) (Yu et al., 2008), three in soybean (*Glycine max* L. *Merr.*) (Luan et al., 2019), as well as additional *HIR* genes in legume (*Lotus japonicus*), cucumber (*Cucumis sativus*), pepper (*Capsicum annuum*), apple (*Malus domestica*), rapeseed (*Brassica napus* L.) and rice (Li et al., 2022; Hakozaki et al., 2004; Kim et al., 2004; Zhao et al., 2019; Jung and Hwang, 2007; Chen et al., 2007). The identification of six *HIR* genes in the *Oryza sativa* genome suggested a diversification of the functions of OsHIR (**Table 1**).

Previous researches shown that most HIR proteins were localized to the plasma membrane (PM) (Choi et al., 2011; Duan et al., 2013; Qi et al., 2011; Wang et al., 2024; Li et al., 2019; Zhao et al., 2019; Zhou et al., 2010). Our findings corroborate this, as we observed that OsHIR proteins were also PM-localized and exhibited the characteristic SPFH domains typical of HIR proteins (**Table 1**, **Figure 1**; **Supplementary Figure S3**). Additonally, the phylogenetic analysis of HIR proteins from *Arabidopsis*, maize, wheat, barley, and rice demonstrated their division into three distinct groups. OsHIR1.3 and OsHIR1.4 were grouped with AtHIR1.1, AtHIR1.2, HvHIR1, HvHIR2, ZmHIR1, and TaHIR2 proteins. On the one hand, OsHIR1.1, OsHIR1.2, OsHIR1.5, ZmHIR3.1, ZmHIR3.2, TaHIR3, and HvHIR3 grouped together as a cluster. On the other hand, OsHIR3, AtHIR3, ZmHIR4, TaHIR4, and HvHIR4 formed another cluster (**Figure 2**). These results indicate a significant conservation level among HIR proteins across different plant species. Our analysis revealed that four out of the six *OsHIR* genes exhibited collinearity (**Supplementary Figure S1**), indicating the notion of evolutionary conservation within this gene family. Furthermore, the lack of collinearity between *OsHIR3* and *OsHIR1.5* with *HIRs* suggests possible evolutionary novelty (**Supplementary Figure S1**). Consistent with an earlier rice genome study (Huang et al., 2012), syntenic maps revealed that *O. sativa* shares a closer evolutionary relationship with *T. aestivum* and *Zea mays* (**Figure 3**). Collectively, these findings indicate that OsHIR proteins may have various activities in rice.

*Cis*-acting elements are particular sequences of DNA that interact with specific transcription factors (TFs) to regulate the expression of downstream genes. This study identified numerous *cis*-acting elements linked to responses to light, anaerobic, hormonal, and stress, located 2.0 kb upstream of *OsHIR* genes (**Figure 5**). In wheat (*Triticum aestivum* L.), *HIR* genes have been proven to respond to environmental stimuli like drought, high salinity, and low temperature (Duan et al., 2013). Here, light responsive elements (LREs), drought responsive elements (DREs), and low temperature responsive elements (LTRs) were also observed in *OsHIR* genes promoters, indicating their roles in abiotic stress response (**Figure 5**). In accordance with previous studies on wheat, apple, and rapeseed (Duan et al., 2013; Zhao et al., 2019; Li et al., 2022), diverse phytohormone response elements, such as the abscisic acid responsive element (ABRE), methyl jasmonate responsive element (MJRE), salicylic acid responsive element (SARE), and gibberellin responsive element (GARE), were found in all *OsHIR* genes promoters (**Figure 5**). These findings suggest that *OsHIR* genes play roles in the signal transduction network for stress response regulation in *Oryza sativa*.

Researches on functional diversity of *HIR* genes in various plant species have shown their roles in stress regulation, such as drought and cold stress responses in wheat (Duan et al., 2013), ABA response in apple (Zhao et al., 2019), and pathogen defense in *Arabidopsis* and other species (Mei et al., 2020; Sun et al., 2021; Wang et al., 2024; Zhao et al., 2019). All *OsHIR* genes exhibited consistent expression under normal conditions (**Figure 6**), highlighting their essential roles in rice. Consistent with the expression of *HvHIRs* in barley and *BnHIRs* in rapeseed (Rostoks et al., 2003; Li et al., 2022), *OsHIR3*, *OsHIR1.2*, and *OsHIR1.3* genes were primarily expressed in leaves, with lower levels in stems (**Figure 6**). Resistance is one of the physiological basis for high crop yield, with leaves serving as primary organs for plant responses. The expression levels of four representative *OsHIR* members (*OsHIR3*, *OsHIR1.2*, *OsHIR1.3*, and *OsHIR1.4*) from three clades under both normal and stress conditions were evaluated to explore their functions in rice. Gene expression level was normalized to unstressed controls, which was set up as ‘1’. The expression patterns of *OsHIR* genes shifted under various abiotic stresses and hormone treatments. In wheat, *TaHIR1* and *TaHIR3* levels dropped during low temperature, drought, and salinity (Duan et al., 2013), a trend we observed in our study (**Figure 7**). PEG treatment down-regulated *OsHIR1.2* and *OsHIR1.3* at 3, 6, and 12 h, while *OsHIR3* was up-regulated twofold at 6 h. Cold stress down-regulated *OsHIR3* and *OsHIR1.3*, whereas *OsHIR1.2* initially decreased then increased. Notably, *OsHIR1.4* increased then decreased under drought and cold stress (**Figure 7**). Previous research has shown raised SA levels in *OsHIR3*-overexpressed rice and *CaHIR1*-silenced plants during *X. campestris* pv. *Vesicatoria* infection, suggesting a potential link between SA and *HIRs* (Li et al., 2019; Choi et al., 2011). The qPCR analysis showed that *OsHIR3* and *OsHIR1.4* were sensitive to all hormonal treatments. Specifically, *OsHIR3* peaked at 3 or 6 h, followed by a decline at 24 h after treatments with ABA, GA, SA, and MeJA. Conversely, *OsHIR1.4* was first down-regulated, then up-regulated by those hormonal treatments (**Figure 7**). It can be speculated that *OsHIR3* and *OsHIR1.4* may have significant roles in response to both abiotic and hormonal stresses. In contrast to reports that ABA down-regulated *TaHIR1* and *TaHIR3*, and MeJA increased *MdHIR4* (Duan et al., 2013; Zhao et al., 2019), we noted that the other *OsHIR* members (*OsHIR1.2* and *OsHIR1.3*) was up-regulated by ABA, down-regulated by GA and MeJA, while unchanged by SA treatment. These observations indicated diverse HIR responses to plant hormones, particularly the defense hormones SA and MeJA. Our findings highlighted that *OsHIRs* from different clades might play varied and dominant roles in maintaining a balance of hormones in plants at different stages of resistance response.

## 5 Conclusion

In conclusion, we identified a total number of six *OsHIR* genes from the *Oryza sativa* genome. Analyses of physicochemical properties, phylogenetics, conserved motifs, gene structure, *cis*-acting elements in promoters, and expression patterns were conducted to understand the functions of *OsHIR* genes. The findings indicate that all four *OsHIR* members tested responded to PEG6000, low temperatures, ABA, SA, MeJA, and GA, highlighting the significant roles of *O. sativa HIR* genes in mediating plant reactions to different abiotic stimuli. This genome-wide identification and characterization of the *HIR* family in *O. sativa* serves as a foundation for further functional studies and for enhancing breeding and genetic improvement in *Oryza sativa*.

## Data Availability

The original contributions presented in the study are included in the article/**Supplementary Material**. Further inquiries can be directed to the corresponding authors.

## Supplementary material

Supplementary Figure 1 Chromosomal distribution and synteny analysis of *OsHIR* genes from *Oryza sativa.* The blue boxes with different lengths labeled as Chr1 to Chr12 stood for the different chromosomes of *O. sativa*. The locations of each *OsHIR* gene from *O. sativa* on the chromosomes were displayed in black. The synteny relationships between different *OsHIRs* were indicated with red lines.

Supplementary Figure 2 Syntenic analysis of *HIR* genes between *Oryza sativa* (rice) and *Arabidopsis thaliana*.

Supplementary Figure 3 Domain prediction of *Oryza sativa* HIR proteins. Green boxes: SPFH domains.
