# Supplementary figures and images for: Genome-wide identification and expression profile of HIR gene family members in Oryza sativa L
Source: Front Plant Sci. 2024 Nov 19;15:1492026. doi: 10.3389/fpls.2024.1492026 (PMC11613742; doi:10.3389/fpls.2024.1492026)

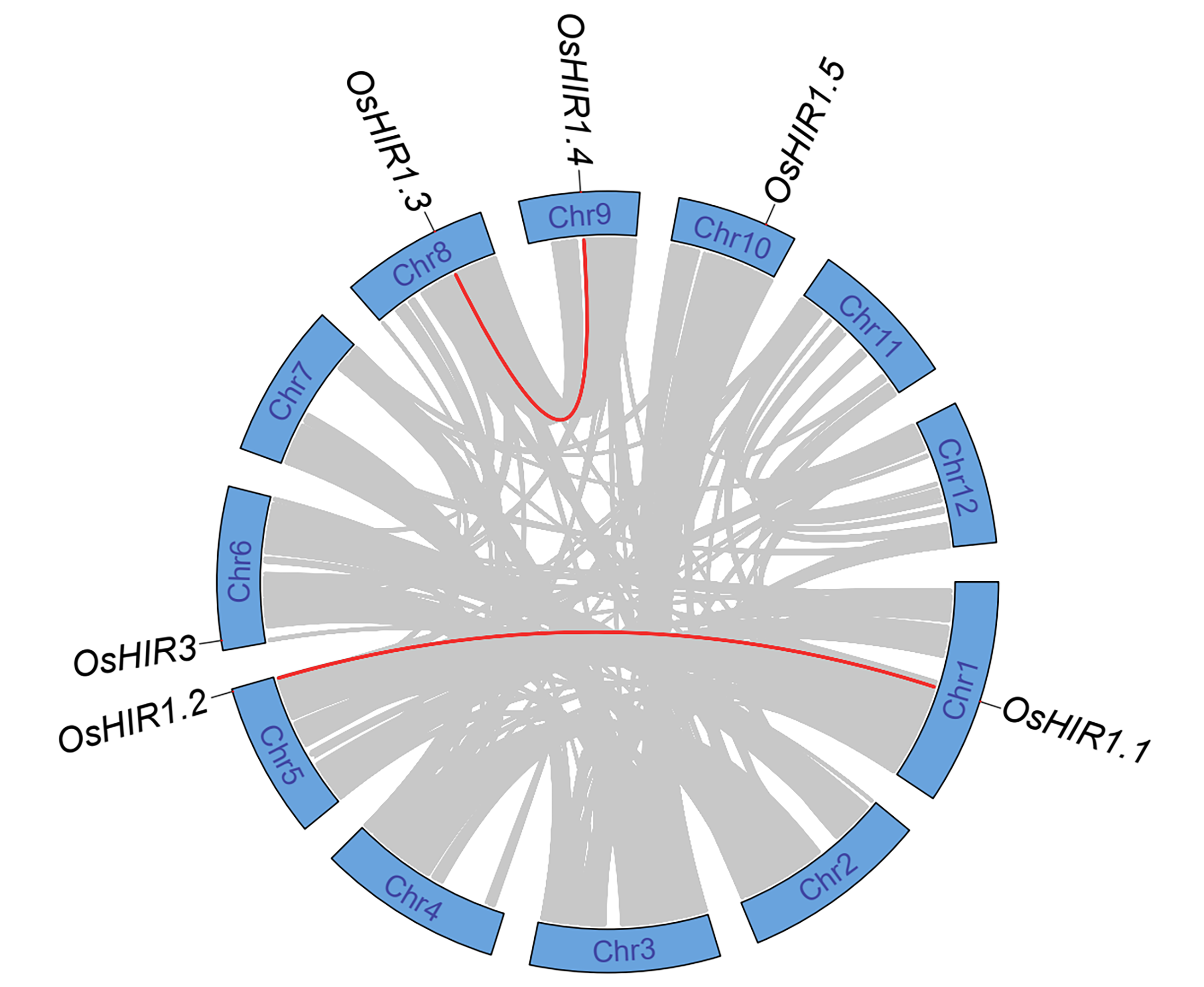

Supplement: Supplementary Figure 1 — Chromosomal distribution and synteny analysis of OsHIR genes from Oryza sativa. The blue boxes with different lengths labeled as Chr1 to Chr12 stood for the different chromosomes of O. sativa. The locations of each OsHIR gene from O. sativa on the chromosomes were displayed in black. The synteny relationships between different OsHIRs were indicated with red lines. [file Image1.tif]

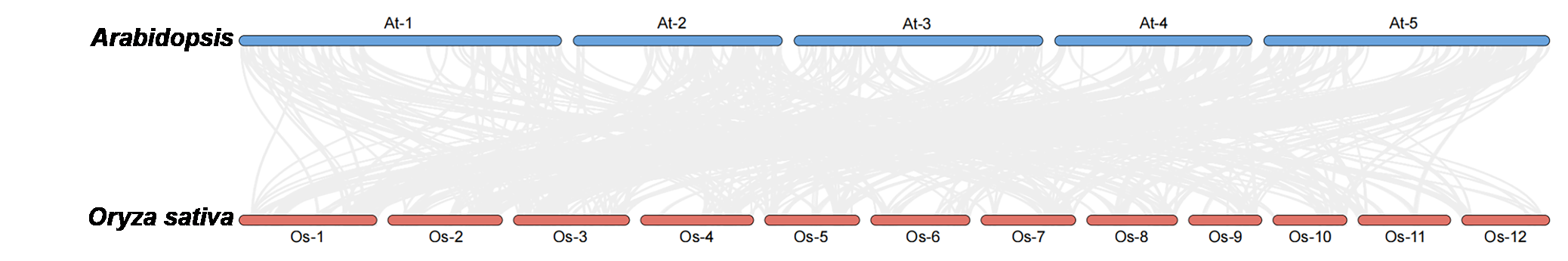

Supplement: Supplementary Figure 2 — Syntenic analysis of HIR genes between Oryza sativa (rice) and Arabidopsis thaliana. [file Image2.tif]

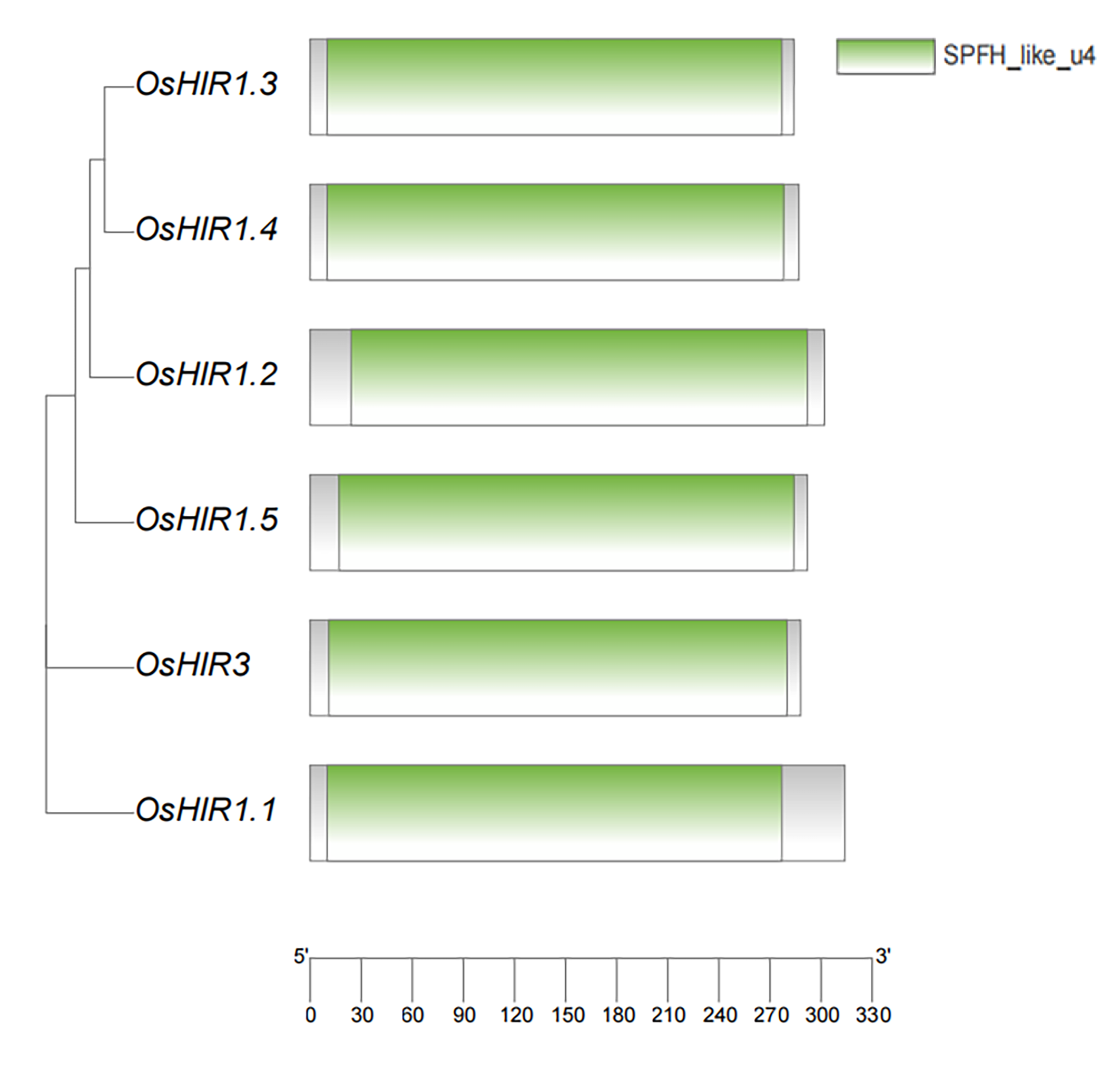

Supplement: Supplementary Figure 3 — Domain prediction of Oryza sativa HIR proteins. Green boxes: SPFH domains. [file Image3.tif]
