# Supplementary material for: Genome-wide identification and expression profile of HIR gene family members in Oryza sativa L
Source: Front Plant Sci. 2024 Nov 19;15:1492026. doi: 10.3389/fpls.2024.1492026 (PMC11613742; doi:10.3389/fpls.2024.1492026)
Supplement: Supplementary file 4 [file Table1.docx]

**Table S1. Primer sequences used for RT-qPCR analysis**

| **Gene name** | **Forward Primer** | **Reverse Primer** |
| --- | --- | --- |
| *OsActin* | GGTATCCATGAGACTACATACAACT | TACTCAGCCTTGGCAATCCACAT |
| *OsHIR1.1* | AAGACCAAGGACAATGTGTAT | GATCTGTGACCTGGTGTTG |
| *OsHIR1.2* | TTCACTAGATAA AGAGGGCG | TTCACTGAGAACCCCAGCAC |
| *OsHIR3* | AAATTCAGGCCTACGTCTTT | ATTTCATTCATTGCTCTGCG |
| *OsHIR1.3* | TGGATGGGCTGAGAGACAGT | GCGATATCTTTGACGGCACC |
| *OsHIR1.4* | CGGAGAATGTCCCTGGAACC | GGGGATGAACACTGAGGTGG |
| *OsHIR1.5* | CTGCGTGCCATGGATTATTGG | CATCGCTTGCTTTGTCCTCC |
